# Too Fresh Is Unattractive! The Attraction of Newly Emerged *Nicrophorus vespilloides* Females to Odour Bouquets of Large Cadavers at Various Stages of Decomposition

**DOI:** 10.1371/journal.pone.0058524

**Published:** 2013-03-13

**Authors:** Christian von Hoermann, Sandra Steiger, Josef K. Müller, Manfred Ayasse

**Affiliations:** 1 Institute of Experimental Ecology, University of Ulm, Ulm, Germany; 2 Department of Evolutionary Biology and Ecology, University of Freiburg, Freiburg, Germany; Institut de Biologia Evolutiva - Universitat Pompeu Fabra, Spain

## Abstract

The necrophagous burying beetle *Nicrophorus vespilloides* reproduces on small carcasses that are buried underground to serve as food for their offspring. Cadavers that are too large to bury have previously been postulated to be important food sources for newly emerged beetles; however, the attractiveness of distinct successive stages of decomposition were not further specified. Therefore, we investigated the potential preference of newly emerged *N. vespilloides* females for odour bouquets of piglet cadavers at specific stages of decomposition. Analyses of walking tracks on a Kramer sphere revealed a significantly higher mean walking speed and, consequently, a higher mean total track length when beetles were confronted with odour plumes of the decomposition stages ‘post-bloating’, ‘advanced decay’ or ‘dry remains’ in comparison with the solvent control. Such a change of the walking speed of newly emerged *N. vespilloides* females indicates a higher motivation to locate such food sources. In contrast to less discriminating individuals this behaviour provides the advantage of not wasting time at unsuitable food sources. Furthermore, in the advanced decay stage, we registered a significantly higher preference of beetles for upwind directions to its specific odour plume when compared with the solvent control. Such a change to upwind walking behaviour increases the likelihood that a large cadaver will be quickly located. Our findings are of general importance for applied forensic entomology: newly emerged *N. vespilloides* females on large cadavers can and should be regarded as potential indicators of prolonged post mortem intervals as our results clearly show that they prefer emitted odour bouquets of later decomposition stages.

## Introduction

During the decomposition process of a cadaver, the occurring volatile organic compounds (VOCs), which are linked in quality and quantity to specific stages of decay [1]–[3], are reliable cues for appropriate succession niches of cadaver-associated insects [4], [5]. For instance, the blowflies *Calliphora vicina* and *Lucilia caesar* (Diptera: Calliphoridae) and also the burying beetles *Nicrophorus vespillo* and *N. vespilloides* (Coleoptera: Silphidae), which are usually amongst the first insect visitors to a cadaver, can detect and orient towards sulfur-containing volatile organic compounds (S-VOCs), such as dimethyl sulfide, dimethyl disulfide and dimethyl trisulfide [5], [6], which are produced by bacteria shortly after the death of an animal.

Our forensic chemo-ecological study focuses on the burying beetle *N. vespilloides*, because, in this species, the question remains open with regard to the preference for odour bouquets of various decomposition stages in its dependency on carcass size. According to its name, the burying beetle *N. vespilloides* buries small vertebrate cadavers in the soil as food for its offspring [7]. Biparental care by one conspecific pair of beetles, which have secured a carcass suitable for reproduction, has been known for a long time in the taxon *Nicrophorus* [8]. The cadaver itself is rolled up under soil into a brood ball, the fur or, in the case of birds, the feathers being mechanically removed [8]. The brood ball is impregnated with anal and oral secretions of the beetles, both secretions of which are known to contain substances that reduce the microbial colonization of cadavers [9], [10]. Hatched larvae are fed by their parents in the form of regurgitated predigested carcass material, with the development of the larvae being completed in only seven days [8] at a temperature of 20°C. In Europe, the seasonal activity of *N. vespilloides* starts early in the season during late April and lasts until September [8], [11]. In April, dense populations of this species emerge and the sexually immature females thereof immediately start with their egg-ripening feeding period as a prerequisite for their reproduction on small carcasses in May [8], [11]. Reproduction on fresh cadavers without any existing infestations of competing carrion-associated species, such as blowflies, and with a low amount of microbial decomposers is highly advantageous [5], [12]. Thus, burying beetles are able to detect a cadaver as early as 1 day post mortem over a distance of up to several kilometres [5], [13].

However, in addition to the above-mentioned ability of fresh carcass detection, cadaver preference in burying beetles appears to depend on the size of the cadaver and the maturity of the beetles [14], [15]. Burying beetles with mature ovaries favour small mice carcasses for reproduction, whereas newly emerged adults with immature ovaries tend to favour large cadavers as an important food source for ovarian development [8], [14], [15]. During the period when ovaries are maturing, dozens of *N. vespilloides* individuals converge on large cadavers that are too big for burial (>300 g, [8], [16]).

In forensic entomology, large insect-inhabited cadavers such as pigs or humans are important study objects for succession-based post mortem interval (PMI) estimations [17], [18]. The entomofaunal succession of a huge richness of carrion-associated species accompanies the decomposition process [19]. In the fresh stage of decomposition, members of Calliphoridae and Sarcophagidae arrive at the cadaver [18]. In the bloated stage (inflated abdomen through gaseous by-products of putrefaction [20]), significant maggot masses can be observed [18]. The post-bloating stage (skin rupture and release of trapped putrefactive gases [20]) is dominated initially by large numbers of feeding fly maggots and predatory beetles such as Staphylinidae and Histeridae [18]. At the end of this stage and also at the beginning of the advanced decay stage (most of the flesh has disappeared, some soft tissue remains in the abdomen [20]), blowfly maggots migrate in intense numbers for pupation [18], [21]. In the last stage of decomposition, namely the dry remains stage, only bones, hair and remains of dried-out skin remain [20].

Matuszewski et al. (2008) conducted a forensic entomological field study with decomposing domestic pig cadavers and found that the number of collected adults of burying beetles peaked in the post-bloating stage of decomposition. An early occurrence of *Nicrophorus* adults was not found, but they were collected until the last day of the study [21]. Peschke et al. (1987) registered the highest peak of *N. vespilloides* in the post-bloating stage of rabbit carcasses. Analogous to the study of Matuszewski et al. (2008), they collected no individuals in the fresh stage of decay, but, in lower abundances, in all the other remaining stages during the entire decomposition period [19].

The findings of the above-mentioned field studies raise the question as to how newly emerged *N. vespilloides* females with immature ovaries can be attracted to the different odour bouquets that occur during the whole course of cadaver decomposition. Therefore, the aim of our forensic chemo-ecological study has been to investigate whether newly emerged *N. vespilloides* females are attracted to the odour bouquets of piglet cadavers and whether they show any preferences for specific decomposition stages (fresh, bloated, post-bloating, advanced decay and dry remains). We collected carcass volatiles of maggot-infested piglet cadavers by means of a headspace sampling technique in the field. We conducted our chemical attraction experiments on a Kramer sphere (‘open loop’ device [22]) in order to find significant differences in the walking tracks and walking parameters of tested burying beetles with regard to distinct offered odour bouquets of piglet cadavers in the above-mentioned five decomposition stages.

## Materials and Methods

### 1. Ethics Statement

All necessary permits were obtained for the described field studies. No animals were killed for this study. Experiments were conducted with stillborn piglets obtained from a local pig farm (Josef Möst, Jedesheim, Germany).

### 2. Rearing of Burying Beetles

Experimental burying beetles, *Nicrophorus vespilloides* were trapped in carrion-baited pitfall traps in a deciduous forest near Freiburg, Germany (48°00′N, 07°51′E). Beetles were reared for 6 generations at the Institute of Experimental Ecology (University of Ulm, Germany). A maximum of four adult beetles of the same sex were kept in moist peat substrate in transparent plastic boxes (100 mm×100 mm×65 mm) in a climate chamber under a 16∶8 light/dark regime, an environmental temperature of 20°C and a humidity of approximately 80%. Decapitated mealworms and mice cadavers (for reproduction purposes) served as a food supply. Shortly after eclosion, the newly emerged female beetles were maintained separately in a climate chamber under a 8∶16 light/dark regime (simulation of short days), an environmental temperature of 15°C and a humidity of approximately 80%. Such rearing parameters are necessary to retard gonad development (Müller, personal observation). At 20°C and under a 16∶8 light regime, *N. vespilloides* is known to become sexually mature after about 14–20 days. Females kept in colder temperatures mature much later. Egg-laying experiments (with a supply of mouse carrion to trigger egg-laying) conducted after 30 short cold days in a climate chamber revealed, in 10 out of 10 cases, no positive oviposition events. This result was regarded as a reliable indication of still-immature gonads, even at 30 days after eclosion. For reliability, exclusively female beetles with ages between 4 and maximal 19 days after eclosion were used for our bioassays.

### 3. Headspace Sampling of Piglet Cadavers

During two consecutive exposure periods, within a fenced grassland in Neusäß (Bavaria, Germany) in summer 2011, we collected 241 headspace volatile samples from a total of 4 piglet cadavers (*Sus domesticus*, 2 kg individual weight). The cadavers were exposed in wire dog cages (63 cm×48 cm×54 cm, Primopet GmbH, Germany) in order to allow insect infestation but to exclude larger scavengers such as crows or foxes. The ambient temperature in the surroundings of a cadaver was logged every 30 minutes with a Voltcraft DL-100T Data Logger (Voltcraft, Germany) mounted inside the wire cage. Volatiles of the first two piglets were sampled daily from June 6, 2011 to July 16, 2011. These two piglets passed through the following 5 stages of decomposition: fresh (days 1–4 post mortem, T_mean_ = 19°C ±8°C); bloated (days 5–7 post mortem, T_mean_ = 17°C ±8°C); post-bloating (days 8–11 post mortem, T_mean_ = 24°C ±10°C); advanced decay (days 12–25 post mortem, T_mean_ = 21°C ±10°C) and dry remains (days 26–40 post mortem, T_mean_ = 22°C ±11°C). The second two piglets were sampled daily from July 25, 2011 to August 28, 2011. These two piglets passed through the following 5 stages of decomposition: fresh (days 1–2 post mortem, T_mean_ = 21°C ±11°C); bloated (days 3–7 post mortem, T_mean_ = 20°C ±8°C); post-bloating (days 8–14 post mortem, T_mean_ = 22°C ±9°C); advanced decay (days 15–22 post mortem, T_mean_ = unknown) and dry remains (days 23–34 post mortem, T_mean_ = unknown). In order to compensate for individual differences in the course of cadaver decomposition, we used two different piglets in each distinct exposure interval.

For the collection of cadaveric volatile compounds, we packed the piglets hermetically into commercial oven bags (Toppits®, 3 m×31 cm extra broad). Incoming air at 100 ml/min was sucked through a charcoal filter (600 mg, Supelco, Orbo 32 large) for cleaning purposes by insertion of a membrane vacuum pump (DC12, FÜRGUT, Aichstetten, Germany). Subsequently, the air passed through the oven bag with the piglet cadaver inside. Over a sampling time of 4 hrs, the exiting air of the oven bag passed through an adsorbent tube in which the volatiles of the carcass were collected in 5 mg Porapak® Q (Waters Division of Millipore, Milford, MA, USA) adsorbent material. For airflow control, a E29-C-150 MM2 sinker flowmeter (Air Products and Chemicals, Netherlands) was used. In order to obtain information about the ever-present environmental volatiles, we used an empty oven bag as a control and collected the volatiles analogously to the above-described conditions (flow rate = 100 ml/min). After the sampling procedure, we used 4×50 µl of a pentane/acetone (9∶1) mixture (Sigma-Aldrich, Munich, Germany, HPLC grade) for the elution of the adsorbed volatile organic compounds. This elution procedure finally yielded sample volumes of approximately 100 µl. For later application of the headspace samples in Kramer sphere bioassays, these samples were stored in hermetically sealed glass ampules at –40°C.

### 4. Recording of Walking Behaviour, Data Processing and Analysis

**Figure 1:**
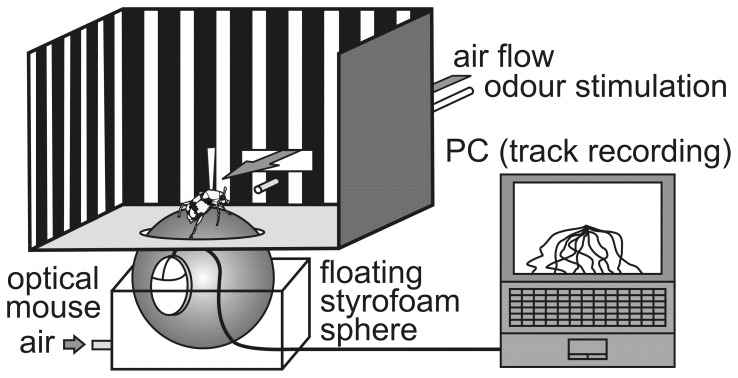
Schematic view of an ‘open loop’ Kramer sphere setup. Movements of the fixed beetle were recorded and traced by an optical mouse. Cadaveric volatile compounds were applied in front of the beetle by an air-streamed Pasteur pipette inside which lay a scent-impregnated piece of filter paper. Via rectangular openings in front of and behind the beetle, a laminar air stream of 50 cm/s was established as the carrier medium of the various applied odour bouquets. The back wall of the arena was only removed for illustration purposes.

After long-distance flights, burying beetles are known usually to land at some remove from a cadaver [5], [13], [23]. Thus, their finally covered walking tracks can be recorded in a bioassay by using a walking beetle on top of a freely rotating ball (a so-called Kramer sphere [22]). The beetle is oriented with its antennae towards an offered scent-loaded air stream (Fig. 1). The movement of the ball indirectly represents the movement of the tested beetle and can be tracked and analysed. We attached the beetles pronotum to a glass bar by a wax-colophony mixture and this bar was vertically mounted above the apex of a freely rotating black-coloured Styrofoam ball (Ø = 8 cm, Fig. 1). Consequently, the beetles were not able to change their head position in relation to the stimulus (‘open loop’ device [24]). However, with their legs and their freely movable abdomen, they were able to move the Styrofoam ball in diverse directions between –90 and +90 degrees from the 0° direction of the stimulus. The Styrofoam ball was floated on an upward-directed air stream. We tracked the locomotion of the beetles by means of an optical mouse that was mounted at the equator of the ball (Fig. 1), 3 mm above its surface [25]. Every 0.5 seconds (sampling interval), we computed, visualized and stored the displacement of the mouse-pointer (position of the burying beetle) in the form of x and y coordinates by means of self-written Microsoft Visual C++ software. For visual control during the tracking procedure, the trajectories of the mouse pointer were visualized on the monitor of a laptop. When the pointer reached the window frame, the software relocated it back to the centre [25] (Fig. 1). The test duration was 5 minutes for each particular run.

For analysis of the walking tracks, we calculated and compared the following 8 walking parameters [26]–[28]: mean walking speed (MWS [cm/s]); mean angular velocity (MAV [°/s]); average length of vectors (ALV [0,1]); length and direction of resultant of all individual mean vectors (AAV [0,1]/°); total track length (TTL [cm]); upwind length (UL [cm]); mean upwind fixation (UF [−1,+1]); mean time spent walking upwind (TSWU [%]).

The 0°-direction was the wind direction and consequently the direction of the odour plumes. Turns to the right were represented as negative angles and turns to the left as positive angles. MWS and MAV were calculated as mean values of 599 instantaneous walking speeds and angular velocities per individual beetle, respectively. The momentary angular velocity represented the velocity of the change in the walking direction between two subsequent sampling intervals. Negative angular velocity indicated clockwise path rotations of the tested beetles. An individual vector originated at the starting point and ended at the final point of an individual run. Its spanning angle described the mean walking direction and its mean length was a quotient of the vector length and the length of the whole distance a tested beetle had actually covered. The value of the mean length ranged between 0 (starting point = end point) and 1 (absolutely straight path). Therefore, ALV was calculated as the average degree of orientation for the tested individual beetles. AAV represented the degree of orientation for the whole population and therefore was calculated as the length and angle (by analogy to ALV) of the resultant vector of all individual mean vectors. TTL was calculated as the mean value of the sums of 599 instantaneously traversed distances per individual beetle. The computation of UL was carried out as the registration of the upwind displacement after the test period of 5 min and served as a measure for the orientation of the individual towards the tested odour plumes. The value of UF as a measure of the degree of direct upwind movement [27] ranged between −1 (absolutely straight downwind movement) and +1 (absolutely straight upwind movement) and was calculated as the quotients of upwind length and total track length per individual beetle. TSWU was calculated as the total walking time in which angles less than 60° or greater than minus 60° from the wind direction (0°) were adopted per individual beetle.

### 5. Provision of Wind and Odour Stimuli

We installed the whole experimental setup inside a laboratory fume hood for a constant air flow supply. A self-constructed cardboard arena (24 cm×24 cm×19 cm) (Fig. 1) with black and white striped walls separated the Kramer sphere from the surroundings in order to avoid optical stimulation of the tested beetles. Additionally, all test runs were performed under red light in order to prevent flight behaviour triggered by artificial light sources. The arena had two rectangular openings (8 cm×3 cm) in front of and behind the mounted beetle in order to permit a constant laminar air stream as the carrier for applied cadaveric volatile organic compounds (Fig. 1). By means of the rectangular opening behind the beetle, we prevented an accumulation of cadaveric volatile compounds inside the arena. The air current velocity inside the arena was 50 cm/s (appropriate value for anemotaxis behaviour [26]), as measured with an anemometer (SKYMATE SM-18, Speedtech Instruments, Virginia, USA) before each single test. To ensure that both beetle antennae were inside the laminar air current, we tested the structure of the odour plumes, previous to our test series, with the smoke of incense cones placed inside the expected laminar air stream. Odour stimuli were provided by an air-streamed Pasteur pipette (inner diameter of 5 mm) under constant flow of 100 ml/min during the entire test duration of 5 minutes. The opening of the pipette (inner diameter of 1 mm) was inserted through a tiny hole (Ø = 2 mm) directly under the rectangular opening in front of the mounted beetle (Fig. 1). The tip of the pipette was positioned at a distance of 12 cm from the beetles antennae and was charcoal precleaned (Alltech Associates Inc., Illinois, USA). Humidified air with a constant flow was maintained by using a membrane vacuum pump (DC12, FÜRGUT, Aichstetten, Germany). For each single test, we placed a wrinkled piece of filter paper (2.5 cm×1 cm) impregnated with 20 µl of test solution (see below) in the inside of the Pasteur pipette.

### 6. Bioassay Procedure and Applied Headspace Samples

Because *N.vespilloides* females typically search for carcasses during the few hours before sunset [29], [30], we conducted our bioassays in a high activity period 2 hours before lights-off in a climate chamber. All beetles were tested at room temperature of about 20°C. Before application of a specific odour bouquet, each beetle was allowed 5 minutes of settling time on top of the sphere. After these 5 minutes, a 20 µl headspace sample diluted 1∶10 with pentane (1/10th of the concentration after 4 hours of sampling time) or 20 µl pure pentane as a control (Table 1) was impregnated on the filter paper by using a micro-syringe (100 µl, Göhler HPLC-Analysentechnik, Chemnitz, Germany). After evaporation of the solvent, the filter paper was introduced into a Pasteur pipette (see above). For the subsequent test period, the walking behaviour of the beetle inside the scent-loaded laminar air stream was recorded. Each beetle was tested in a random order against maximally four (in order to reduce tiring) of the following six test samples: pentane (solvent control); fresh; bloated; post-bloating; advanced decay; dry remains (Table 1). Between two consecutive test samples, the beetles were allowed a 5 minute resting time in a scentless laminar air flow. In order to avoid learning effects, each individual beetle was only tested once with the same test odour bouquet. If an individual walked less than 4 metres in the 5-minute test period, it was discarded. The maximal traversed walking distance was 35 metres and the average traversed walking distance was 22 metres plus/minus 6 metres.

**Table 1:** Headspace samples used in the Kramer sphere bioassay.

Test	Exposure time before sampling	Exposition period	Mean
samples	[days p.m.^a^]	summer 2011	temperature [°C]
fresh	2	06.06.–16.07.	21
	1	25.07.–28.08.	18
bloated	6	06.06.–16.07.	18
	7	06.06.–16.07.	18
	5	25.07.–28.08.	20
post-bloating	8	06.06.–16.07.	18
	8	06.06.–16.07.	18
	8	06.06.–16.07.	18
advanced	16	06.06.–16.07.	20
decay	19	06.06.–16.07.	19
dry remains	30	06.06.–16.07.	21
	31	06.06.–16.07.	21

Test samples are representative of the bouquet of distinct stages of decomposition of piglet cadavers. Each sample was collected for 4 hrs. The mean temperature is the mean value of the recorded temperature values during the 4 hrs of headspace sampling.

a days post mortem (p.m.).

### 7. Statistics

The responses of newly emerged *N. vespilloides* females to various odour stimuli were compared by using a multivariate general linear model (GLM) with odour as the fixed factor and MWS, MAV, ALV, TTL, UL, UF and TSWU as dependent variables. Levene’s test of equality of error variances revealed homogeneous variances for all dependent variables (all P>0.2). Computed walking parameters with significant effects in the model were further analysed with Tukey’s honest significant difference (HSD) post hoc test (significance level = 0.05) to localize the significant differences between the five distinct test samples (Table 1) and the solvent pentane. All statistical analyses were performed by using SPSS (Version 19, IBM, USA).

## Results

The type of the presented headspace sample had an effect on the walking parameters (dependent variables) recorded (F_30_ = 1.741, P = 0.009). GLM tests of between-subjects effects showed that decomposition odour significantly affected MWS (F_5_ = 3.993, P = 0.002), MAV (F_5_ = 2.292, P = 0.047), TTL (F_5_ = 3.993, P = 0.002) and UL (F_5_ = 2.701, P = 0.021), whereas decomposition odour had no significant affect on the parameters ALV (F_5_ = 0.427, P = 0.830), UF (F_5_ = 0.363, P = 0.873) and TSWU (F_5_ = 1.196, P = 0.312).

**Figure 2:**
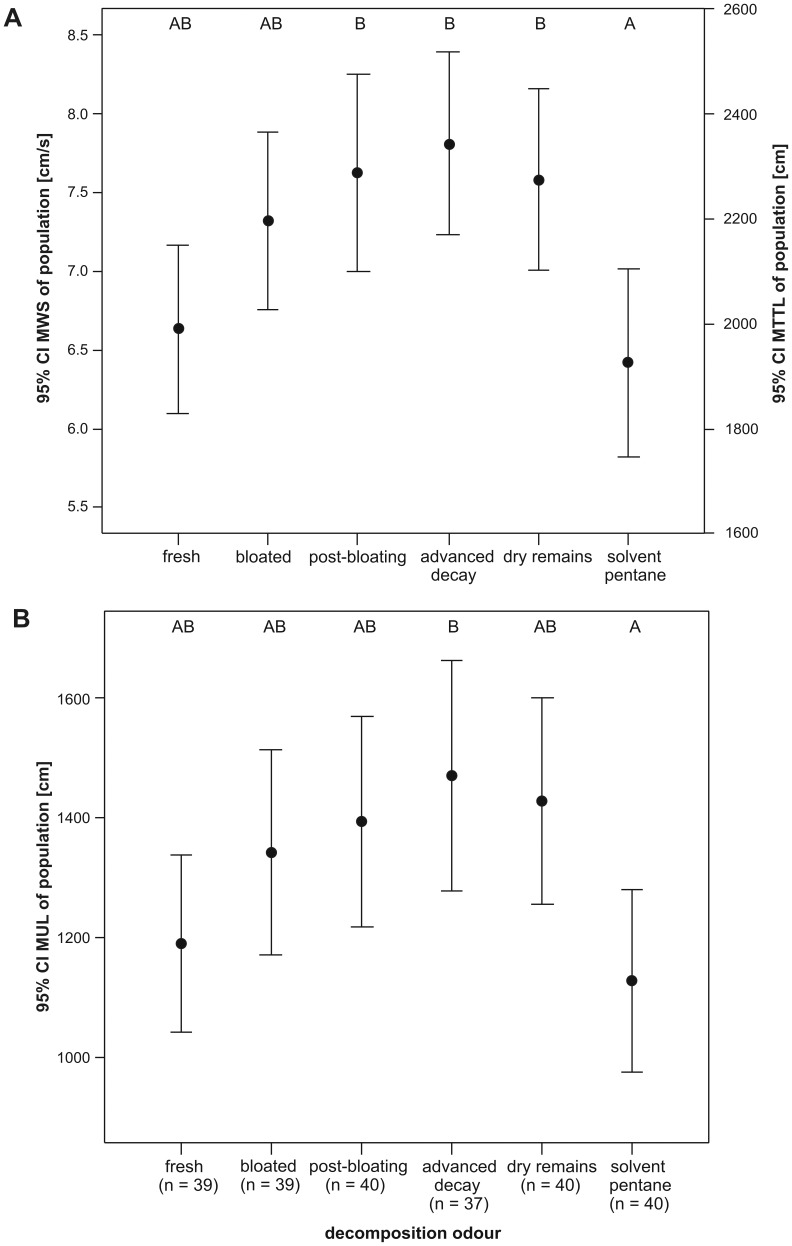
Attractiveness of various decomposition stages of piglet cadavers. **A** Walking speed (MWS; left y axis) and total track length (TTL; right y axis) and **B** Upwind length (UL) of female burying beetles (n = sample size) in relation to odour bouquets from five different decomposition stages and the pure solvent. The mean (M) and the 95% confidence interval (CI) are shown for each odour bouquet. Distinct letters label significant differences (GLM, Tukey’s HSD test, P<0.05).

MWS and consequently also TTL were significantly higher in the decomposition stages of post-bloating, advanced decay and dry remains in comparison with the pure solvent (Fig. 2A and Table 2). Additionally, in the advanced decay stage, the measure of beetle orientation towards its respective odour plume (UL) was significantly higher when compared with the supply of pure solvent (Fig. 2B and Table 2).

**Table 2:** Comparison of walking parameters for the various samples tested in the Kramer sphere bioassay.

Test samples	Numberof runs	Mean walking speed (MWS)	Total track length (TTL)	Upwind length(UL)
	N	*V* [cm/s]	[cm]	[cm]
pentane	40	6.42±1.87	1924.36±561.64	1125.95±479.39
fresh	39	6.63±1.66	1989.87±496.39	1188.82±459.67
bloated	39	7.32±1.74	2196.51±523.30	1341.99±531.91
post-bloating	40	**7.62** a ±1.96 *	**2287.17** a ±586.83 *	1392.40±551.80
advanced decay	37	**7.81** b ±1.75 *	**2343.48** b ±523.50 *	**1470.36** c ±577.58 *
dry remains	40	**7.59** d ±1.80 *	**2275.46** d ±538.45 *	1427.96±536.04

With the exception of the values of parameter AAV, mean values and the 95% confidence intervals (CI) are shown. Bold numbers indicate significant differences compared with the solvent pentane (Tukey’s HSD post hoc tests, * P<0.05).

a P = 0.034.

b P = 0.01.

c P = 0.049.

d P = 0.045.

**Figure 3:**
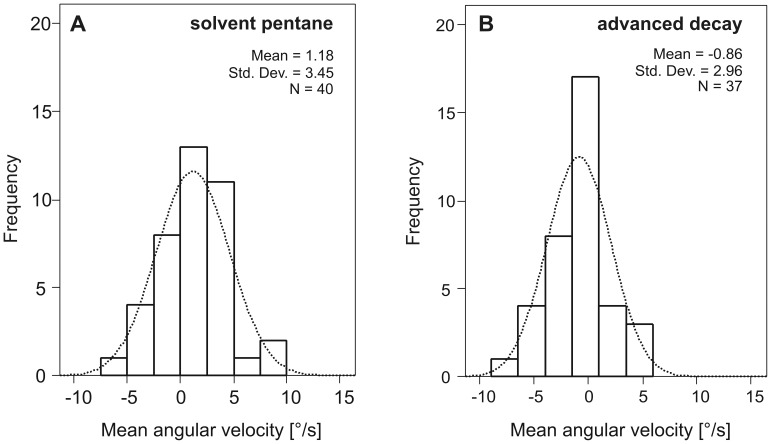
Frequency distributions of the mean angular velocities (MAVs) in relation to two different odour plumes. **A** solvent pentane; N = 40 walks and **B** advanced decay; N = 37 walks. The stimulus comes from the 0° direction. The dotted lines outline the normal distribution curves (Std. Dev. = standard deviation).

As mentioned above, decomposition odour affected the MAV of females (F_5_ = 2.292, P = 0.047) but the post-hoc tests revealed no significant differences in the angular velocities between the six different odour bouquets (Tukey HSD, all P>0.06, Table 3.) However, the frequency distribution of the MAV showed, tendentially, a sharper peak (better orientation) and a smaller scatter (higher running smoothness) in the case of beetle antennae stimulation with advanced decay odour (scatter ±2.96°/s, Fig. 3B and Table 3 when compared with the stimulation with the pure solvent (scatter ±3.45°/s, Fig. 3A and Table 3.) This result is in accordance with the highest mean upwind length and consequently the best orientation of beetles to decomposition odour in the advanced decay stage (mean_UL_ = 1470 cm, Table 2, Fig. 2B and Fig. 3B).

**Table 3:** Comparison of walking parameters for the various samples tested in the Kramer sphere bioassay.

Test samples	Numberof runs	Mean angular velocity (MAV)	Average length of the vector (ALV)	Length/direction of resultant mean vector (AAV)	Upwindfixation (UF)	Time spent walking upwind(TSWU)
	N	*omega* [°/s]	*l*	*l*_r_/*phi*		[%]
pentane	40	1.18±3.45	0.60±0.16	0.96/−4.39°	0.57±0.17	60.53±18.52
fresh	39	−0.87±3.53	0.60±0.15	0.98/−2.10°	0.59±0.15	61.10±16.27
bloated	39	0.33±3.23	0.62±0.15	0.98/−2.34°	0.60±0.15	64.74±17.26
post-bloating	40	0.32±3.62	0.62±0.16	0.97/−1.43°	0.60±0.17	63.45±18.92
advanced decay	37	−0.86±2.96	0.63±0.15	0.98/2.56°	0.61±0.15	67.71±16.84
dry remains	40	0.24±2.70	0.64±0.13	0.97/−6.93°	0.61±0.14	67.35±15.89

With the exception of the values of parameter AAV, mean values and the 95% confidence intervals (CI) are shown.

With regard to the degree of direct upwind movement, no difference was seen between the six distinct offered odours (UF in Table 3.) The same was true for the mean TSWU (Table 3.) Angles of less than 60° or greater than –60° from the 0° direction of wind and odour supply were adopted between 60% and 68% of the total walking time by all analysed burying beetle populations (TSWU in Table 3.).

**Figure 4:**
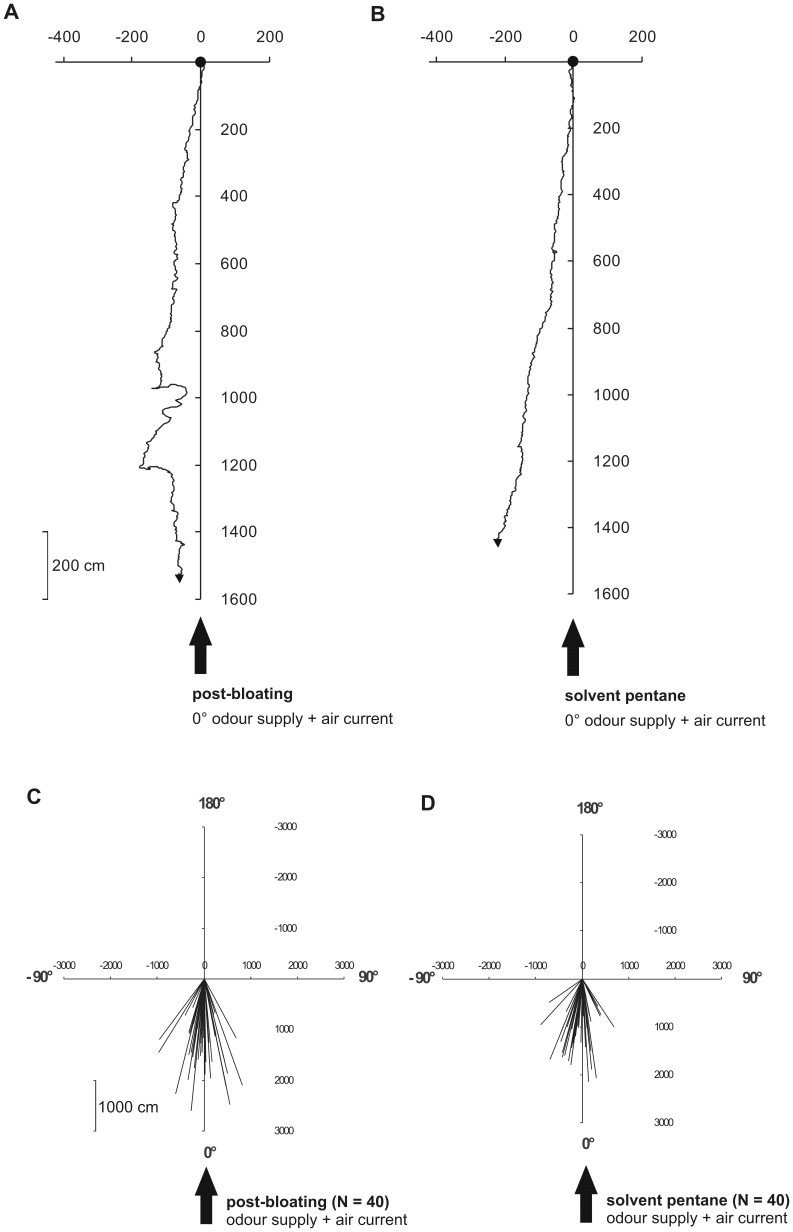
Representative walking tracks of individual female beetles. Provision of headspace samples of **A** a post-bloated piglet cadaver and of **B** pentane solvent. The black dots mark the starting points of the respective 5-min runs and the black arrowheads indicate the endpoints and walking directions. Representative vector diagrams of the mean walking directions of females (N = 40) when headspace samples of **C** a post-bloated piglet cadaver and of **D** pentane solvent were provided. Black arrows denote the common direction (0°) of wind and odour stimuli. Each tested individual beetle was mounted with its head upwind against the 0° direction.

The lengths of the resultant mean vectors (AAV) of all tested burying beetle populations showed a high degree of orientation relating to the six different offered test odours (all *l*_r_ ≥0.96, Table 3 and corresponding vector diagrams in Fig. 4C and D). The directions of these vectors also indicated a strong preference of beetles for the 0° direction of wind and odour stimuli (all angles *phi* between –7 and +3 degrees, Table 3, Fig. 4C and D).

Correspondingly, during the 5 minutes of test duration in an air current of 50 cm/s plus a specific supplied odour plume, walking *N. vespilloides* individuals generally exhibited relatively straight and stable courses (ALV, all *l* ≥0.6, Table 3), independent of the nature of the offered odours (Fig. 4A and B), and therefore showed so-called anemotactic behaviour [26]. However, as mentioned above, the total track length depended on the nature of offered odours, as is also depicted in an exemplary form in Fig. 4A and B. When the odour of a post-bloated piglet was offered, a higher total track length (22.73 m) was achieved than when the pure solvent was provided (17.34 m; Fig. 4A and B). Decomposition odour changed the wind orientation behaviour of the exemplary female in such a way that zigzag subcourses against the wind were the result (Fig. 4A). However, after a test duration with a decomposition odour supply for 5 minutes, the walking direction of the specific female was more upwind-oriented compared with the test situation with pentane (Fig. 4A and B).

## Discussion

### Discrimination between Stages of Decomposition of Large Vertebrate Cadavers in Newly Emerged N. vespilloides Females

In 1984, Wilson and Knollenberg detected newly emerged females of *Nicrophorus tomentosus*, *N. orbicollis* and *N. defodiens* with immature ovaries in baited pitfall traps that simulated a high concentration of carrion volatiles as is typical for large cadavers. In addition, they demonstrated that females with mature ovaries avoided large cadavers but showed, instead, a preference for small cadavers that are suitable for burying and reproduction. However, they had no clear explanation for the underlying proximate mechanisms of discrimination, such as different preferences for different chemicals (quantitative or qualitative) in odour bouquets of differently decomposed cadavers of various sizes [14].

In the current study, we have started to explore the underlying proximate mechanism; we have investigated whether immature females of *N. vespilloides* show any behavioural response towards the odour of large cadavers and determined which stage of composition they prefer. Our results suggest that newly emerged burying beetles females respond to and are able to discriminate between the odour bouquets of various decomposition stages of large cadavers: only the stages of post-bloating, advanced decay and dry remains lead to a significant increase of the mean walking speed, and not the fresh and bloated stages. Such a chemically triggered change of their walking speed indicates a higher motivation to locate such food sources. In contrast to less discriminating individuals this behaviour provides the advantage of not wasting time at unsuitable food sources. Our detected behaviour in walking beetles could probably be considered as congruent to the decisions made by burying beetles in flight. A flying beetle that does not waste time investigating a fresh cadaver would have a clear advantage over a less discriminating beetle, because, on freshly dead small cadavers (enough carcass material for one female’s brood to survive [8], [31], [32]), predation, fights or poor feeding might occur. On unburied large cadavers such as piglets like in our study (feeding substrate for ovarian development as a prerequisite for reproduction), newly emerged burying beetles as early feeders should have a fitness advantage in competition with large numbers of other carcass-associated insects (possibility of rapid consumption of whole cadaver tissue by necrophagous flies) or vertebrate scavengers. Most likely, the perception of such a valuable large food source increases the beetles’ motivation and consequently their willingness to invest the larger amount of energy that is needed for faster movement. Another consequence of a higher walking or flight speed is shown in the eucalyptus woodborer *Phoracantha semipunctata* (Coleoptera: Cerambycidae). In this species, a faster flight speed is coupled with path linearity and a lower turning rate in the case of permanent contact with an odour plume [33]. In *Nicrophorus humator*, path linearity increases the travelled distance between the starting and endpoint (and consequently the range of the explored environment) from usually 1 m in a windless bioassay environment to 9 m in an air current of 100 cm/s (5 minutes of walking time on a locomotion compensator; [26]). These aspects are especially important for the burying beetle, which has to detect cadavers over distances of up to several kilometres [5], [13]. Higher mobility probably increases the chance for cadaver detection.

From a forensic entomological point of view, we find it interesting that newly emerged *N. vespilloides* females with immature ovaries show a strong preference for the odour bouquets of later stages of decomposition (from post-bloating over advanced decay to dry remains; days 8–31 post mortem, T_mean_ = 19°C) of large cadavers. More precisely, these females show not only a higher walking speed, but also a tendency to higher running smoothness and the highest orientation towards odour plumes of decomposed cadavers in later stages. These findings are also supported by several succession and decomposition-based field studies. Peschke et al. (1987) performed extensive field investigations with rabbit carcasses of approximately 2800 g in weight, similar to the weight of our piglet cadavers, in Bavaria in Germany (the same federal state as in our study) from 1976 to 1983. In accordance to the preferences found in our study, they registered the highest abundance of *N. vespilloides* in the post-bloating stage and they collected no individuals at the fresh stage of decay [19]. In the remaining decomposition stages (bloated, advanced decay and dry remains), they also collected *N. vespilloides* individuals but with lower abundances compared with the post-bloating stage [19]. Matuszewski et al. (2008) performed forensic entomological field studies to determine insect succession and carrion decomposition in various forest habitats of western Poland. They used domestic pig cadavers of a mean weight of 34 kg as adequate models for human corpses. *Nicrophorus* adults could be collected right up until the last day of the study with the highest abundance in the post-bloating stage [21]. Matuszewski et al. (2008) also stated that the early occurrence of adult *Nicrophorus* species was not found in decomposition studies with large cadavers, a finding that agrees with our results from the tracking analysis.

A possible explanation for our findings and also for the observations of the cited studies could be that newly emerged burying beetle females with immature ovaries prefer large cadavers in order to feed on blowfly maggots [8], [11], [15], [21], [34], [35]. This is supported by the results of a field study of Kentner and Streit (1990) with 9 exposed rat cadavers in various biotopes. They stated that adult *Nicrophorus* species are predators and feed only rarely on decomposed meat. The authors concluded that adult burying beetles are also attracted by older cadavers where they feed upon fly maggots [15]. The preference of odour bouquets emitted by large cadavers in later stages of decomposition, such as the post-bloating or the advanced decay stage might help burying beetles to detect suitable feeding sites, as the dominance of feeding and migrating blowfly larvae is the highest in these stages of decay [21]. In the post-bloating stage, masses of maggots have been found to feed on the soft tissues of a cadaver and, in the initial advanced decay stage, an intense migration of maggots can be observed [18], [21]. Blowfly larvae excrete urea and allantoin, which give the breeding substrate a characteristic intense smell. The antimicrobial properties of urea and allantoin cause a reduction in the microbial decomposition of the corpse [36], [37], which additionally affects the odour bouquets of cadavers and consequently influences the specific scent attraction of carcass-associated insects such as the burying beetle. During our headspace sampling procedure in the field, we included blowfly maggots and offered the complete odour bouquets of maggot-infested piglet cadavers in our tracking experiments. We detected a dominance of dipteran larvae in the post-bloating stage and the migration of post-feeding L3-larvae (third instar) in the advanced decay stage during our field work (headspace sampling) in this study. The odour bouquets of the two stages with the highest dominance of feeding and migrating blowfly larvae (a good food source for female burying beetles) elicited a higher mean walking speed of the beetles in the tracking experiments.

The attractiveness of cues from cadavers with substantial blowfly maggot populations indicates that these cadaver inhabitants are of major importance in the diet of burying beetles. Further studies will be needed to clarify whether newly emerged burying beetles seek out large cadavers mainly to feed on fly larvae (as assumed by Kentner and Streit (1990), see above) or whether they also feed directly on cadaver substrate. From a phylogenetic point of view, the majority of carrion beetles (Coleoptera: Silphidae) are known to feed on cadavers of either vertebrates or invertebrates [38]. Only the more derived taxa *Ablattaria*, *Dendroxena* and *Phosphuga* are highly specialized predators of snails or caterpillars [39]–[41]. If *Silphidae* and *Staphylinidae* are sister taxa [42] then their last common ancestor might have been a predator of fly maggots, because many staphylinids live predaceously on fly larvae.

Interestingly, the odour of the dry remains stage, i.e. the period at which arthropod activity has almost ceased, also raised the mean walking speed of *N. vespilloides* females. Electrophysiologically active (EAD-active, ‘smellable’) compounds might be present in higher quantities in a decomposition stage that only consists of hardened skin and bones than in earlier decomposition stages (von Hoermann, unpublished data) and therefore could modify the beetles behaviour. Future consideration of available cues (constraints of sensory detection) versus adaptive behaviour might aid our understanding of the response to dry remains odour. It is possible, that newly emerged females are not able to perceive fresh cadavers. In that case constraints in sensory detection rather than adaptation explain why young females respond to later decomposition stages in our experiments. Examining the olfactory capabilities of burying beetles and the chemical composition of cadaver odours will help to determine if sensory constraints are responsible for our observations. Currently, we are conducting GC-EADs (gas chromatography coupled with electroantennographic detection) with the antennae of newly emerged *N. vespilloides* females and chemical analysis (coupled gas chromatography-mass spectrometry (GC-MS)) in order to identify the patterns of behaviourally active cadaveric VOCs in this species over time (von Hoermann, in preparation).

### Orientation of Newly Emerged N. vepilloides Females in Decomposition Odour-loaded Air Streams

Our results show that newly emerged *N. vespilloides* females exhibit a typical anemotactic behaviour in a constant air current of 50 cm/s. All tracked courses are relatively straight and stable, regardless of which specific odour bouquet is offered. Heinzel and Böhm (1989) stated that such a general wind-orientation behaviour could improve the search of odour plumes (and consequently the cadaver itself) in the case of a possible loss of contact during the landing procedure at some distance to the cadaver. This proposed explanation is in accordance with our finding that pure solvent in combination with an air current (analogous to contact lost of odour plumes) also arouses wind-orientation. The wind-oriented straight walking behaviour in air currents of 50 to 150 cm/s has previously been demonstrated for another burying beetle species, *Nicrophorus humator* [26]. In a windless environment, on the other hand, this species shows an inherent internal turning tendency [26]. When we look more in detail at the walking characteristics of individual *N. vespilloides* tracking paths, we can find zigzag-shaped walking reactions (and therefore higher total track lengths) in air-current fields loaded with decomposition odour bouquets. The same is true for *N. humator* in an air current with additional added carrion odour in the form of successively offered pulses lasting 0.85 seconds [43]. Such behaviour is also well-known from moths while searching for pheromone sources [44], [45]. Moths sense the overall shape of pheromone plumes during zigzag flights, by flying in and out of the plume borders in an alternating manner (“aerial trail following”, [45]). Similar chemo-orientation mechanisms are also known in walking ants that follow ground-deposited pheromone trails [46]. Therefore, in the burying beetle *N. vespilloides*, successive comparisons of the positions of decomposition odour plumes in combination with wind-orientation [26] might enable them to walk along the plume’s long axis towards the cadaveric resources [47].

In locomotion compensator experiments, Böhm and Wendler (1988) found that *N. humator* measures the actual air stream and the actual concentration of carrion volatiles. They stated that the integration of both inputs is necessary for the insect to reach an appropriate odour source by means of wind-orientation, as they were able to demonstrate that, even after one odour stimulus, the wind-orienting behaviour of *N. humator* could be changed and the walking direction could be directed more upwind after the whole odour stimuli period [43]. Because *N. vespilloides* females also exhibit wind-oriented walking tracks with zigzag-shaped structures and a more upwind orientation at the end of a 5-minute run, we conclude that this statement is also valid in this species.

### Conclusions

In a forensic chemo-ecological approach with a highly sensitive ‘open-loop’ tracking system, we tested the preference of newly emerged *N. vespilloides* females with immature ovaries for odour bouquets of large cadavers at five different decomposition stages. We have been able to show that sexually immature females prefer odour bouquets of large cadavers only when they are in later stages of decomposition (from post-bloating over advanced decay to dry remains; days 8–31 post mortem, T_mean_ = 19°C). We assume that volatiles of large numbers of blowfly maggots in combination with cadaveric odour bouquets are responsible for this phenomenon in the necrophilous and predacious species *N. vespilloides*.

Additionally, our study indicates that immature *N. vespilloides* females show zigzag-shaped walking reactions inside relatively straight wind-oriented tracking paths as a search strategy for reaching large cadavers, as previously discussed for Nicrophorinae in *N. humator* [43].

This is the first study in which the attraction of newly emerged *N. vespilloides* females to headspace samples of maggot-infested piglet cadavers has been investigated during an entire decomposition period. At present, we are studying qualitative and quantitative differences of EAD-active volatiles at the various decomposition stages of maggot-infested large cadavers. Electrophysiological experiments with *N. vespilloides* antennae and subsequent GC and GC-MS analysis should much improve our knowledge about the nature of the substances responsible for the preference of later decomposition stages.
